# GPR34 Stabilized by Deubiquitinase USP8 Suppresses Ferroptosis of ATC

**DOI:** 10.1155/mi/5576056

**Published:** 2025-08-18

**Authors:** Bokang Yan, Jiaxing Guo, Meiyuan Huang, Zhecheng Li, Jingyue Sun, Hailong Tan, Weiwei Lai, Shi Chang

**Affiliations:** ^1^Institute of Large-Scale Scientific Facility and Centre for Zero Magnetic Field Science, Beihang University, Beijing 100191, China; ^2^National Clinical Research Center for Geriatric Disorders, Xiangya Hospital, Central South University, Changsha 410008, Hunan, China; ^3^Department of Pathology, Zhuzhou Hospital Affiliated to Xiangya School of Medicine, Central South University, Zhuzhou 412007, Hunan, China; ^4^Department of Hematology, Zhuzhou Hospital Affiliated to Xiangya School of Medicine, Central South University, Zhuzhou 412007, Hunan, China; ^5^Department of General Surgery, Xiangya Hospital, Central South University, Changsha 410008, Hunan, China; ^6^Department of Pathology, Xiangya Hospital, Central South University, Changsha 410078, Hunan, China; ^7^Hunan Provincial Key Laboratory of Pediatric Orthopedics, The Affiliated Children's Hospital of Xiangya School of Medicine, Central South University, Changsha 410007, Hunan, China; ^8^Clinical Research Center for Thyroid Disease in Hunan Province, Changsha 410008, Hunan, China

**Keywords:** ATC, DUB-IN-3, ferroptosis, GPR34, USP8

## Abstract

G protein-coupled receptor 34 (GPR34) is an orphan receptor within the G protein-coupled receptor (GPCR) superfamily, and its specific role in anaplastic thyroid carcinoma (ATC) remains to be elucidated. In this study, we observed that GPR34 was aberrantly upregulated in ATC and the deletion of GPR34 inhibited tumor progression both in vivo and in vitro. Additionally, suppression of GPR34 promoted ferroptosis in ATC cells. We further identified USP8 as a deubiquitinase (DUB) for GPR34, and the effects induced by GPR34 deletion were reversible through USP8 overexpression. Moreover, targeting USP8 with the inhibitor DUB-IN-3 effectively restrained ATC growth. Together, the present study revealed the role of GPR34 in ATC progression and ferroptosis, discovered its corresponding DUBs, and proposed GPR34 as a promising target for ATC therapy.

## 1. Introduction

Anaplastic thyroid carcinoma (ATC), also referred to as undifferentiated carcinoma, arises from the follicular cells of the thyroid gland and represents an extremely malignant subtype of thyroid cancer (THCA) characterized by a rapid growth rate and high invasion [1–3]. Although ATC constitutes merely 2%–5% of all THCA cases, it accounts for 33%–50% of THCA-related mortality [4, 5]. Patients diagnosed with ATC exhibit a median survival time of only 3–7 months, with more than 40% of patients developing distant metastases [6, 7]. Given its inherent resistance to both radioactive iodine and conventional chemotherapy, there are currently no effective surgical or chemoradiotherapeutic interventions that significantly prolong patients' overall survival [8–10]. Consequently, it is imperative to elucidate the mechanisms underlying the initiation and progression of ATC.

Ferroptosis, a recently identified form of iron-dependent nonapoptotic cell death, is characterized by dysregulated cysteine and glutathione metabolism and the accumulation of iron-dependent peroxidized lipids [11, 12]. This intricate process is governed by many cellular metabolic pathways, including redox homeostasis, mitochondrial function, and various signaling pathways [13, 14]. Increasing studies indicates that ferroptosis plays a pivotal role in the regulation of various tumors and holds promise as a potential strategy for cancer therapy [15, 16]. Several researches have suggested that ferroptosis regulators may be involved in the repression of THCA [17]. For instance, inhibition of solute carrier family 7 member 11 (SLC7A11), an essential ferroptosis suppressor, could alleviate PTC (papillary thyroid cancer) progression [18]. Additionally, inactivation of glutathione peroxidase 4 (GPX4) by vitamin C treatment may contribute to ATC growth inhibition [19]. A reduction in CD71 levels in ATC cells has been associated with an increased tolerance to iron overload, thereby enhancing resistance to ferroptosis [20]. However, it is still largely unexplored whether other potential proteins may regulate ferroptosis and involved in the malignant progression of ATC.

G protein-coupled receptor 34 (GPR34) is a 7-transmembrane orphan receptor, belonging to the GPCR family, which affects multiple biological and physiological functions, such as cell proliferation and motility, immune infiltration, gene transcription, etc., and targeting GPR34 is considered a promising disease treatment strategy [21, 22]. Growing studies determine that the upregulation of GPR34 is implicated in the onset and progression of various cancers, including triple negative breast cancer (TNBC), cervical cancer, colorectal cancer (CRC), MALT lymphoma, gastric cancer, glioma, and BCR/ABL-positive leukemia [23–29]. Several researches shows that GPR34 plays a critical role in regulating apoptosis [26, 30, 31], for instance, GPR34 knockout could inhibit ERK1/2 phosphorylation while promoting the activation of caspase-3 to stimulate apoptosis of dendritic cells [30]. However, the tumorigenic role of GPR34 in ATC and its specific mechanism remain unclear, and whether GPR34 has regulatory value in other types of programed cell death (PCD) is uninvestigated.

It has been reported that the activity of GPR34 is regulated by posttranscriptional regulators microRNAs (miRNAs) including miRNA-381/miRNA-300, and NF-κB and MAPK pathways are also involved in the downregulation of GPR34 [24, 31]. Increasing studies indicate that posttranslational modifications including deubiquitination controls the expression of GPRs [32], such as, USP24 stabilizes GPR31 through deubiquitination in nonsmall cell lung cancer (NSCLC) [33], and β2 adrenergic receptor (β2AR) is deubiquitinated by USP33 and USP20 on late endosomes [34]. However, the deubiquitinases (DUBs) charged for GPR34 stabilization in ATC remains unidentified.

Therefore, this leads us to explore the carcinogenic effects of GPR34 on ATC and its specific regulatory mechanisms, and further elucidate the impact of GPR34 on cell fate, which may provide a new effective target for cancer therapy.

## 2. Materials and Methods

### 2.1. Cell Culture, siRNAs, Plasmids, and Chemicals

The human thyroid cell line (Nthy-ori 3–1) and ATC cell lines (BHT101, 8305c, and FRO) were acquired from the Institute of Medical Sciences at Xiangya Hospital. Cells were maintained in DMEM medium (GIBCO, Rockville, MD, USA) containing 10% fetal bovine serum (Pricella Biotechnology Co., Ltd, Wuhan) and penicillin/streptomycin, and grown in an incubator at 37°C with 5% CO_2_.

GPR34-siRNA was purchased from Sangon Biotech Co., Ltd, Shanghai. USP8 and USP33 overexpression plasmids were gifts from Professor Yongguang Tao,and using Lipofectamine3000 (Invitrogen), we transfected the plasmid and siRNA into ATC cells.

Ferroptosis inducer RSL-3 (T3646) and ferroptosis inhibitor ferrostatin-1 (Fer-1) (T6500) were purchased from TOPSCIENCE. Apoptosis inducer VCR (S1241), autophagy inducer Rapamycin (S1039), and pyroptosis inducer LPS (S7850) + Nigericin (S6653) were provided by Selleck. DUB-IN-3 (HY-50737-11866) were obtained from MedChemExpress.

### 2.2. RT-qPCR

PrimeScriptTM RT reagent kit with gDNA Eraser (Takara, Kusatsu, Japan) was used to synthesize cDNA from total RNA isolated using TRIzol (Takara, Kusatsu, Japan). ABI 7500 and FastStart Universal SYBR Green Master were utilized to conduct RT-qPCR. Gene expression levels were normalized using GAPDH. The sequences of RT-qPCR primers are provided in Table S1.

### 2.3. Western Blot Analysis and Co-Immunoprecipitation (Co-IP) Assay

The collected cells were lysed using a protease inhibitor cocktail-containing RIPA lysis buffer. Utilizing SDS-polyacrylamide gel, proteins were separated, then transferred to PVDF membranes. Primary antibodies included GPR34 (YT2014, Immunoway), GAPDH (60004-1-Ig, Proteintech), USP8 (67321-1-Ig, Proteintech), USP33 (20445-1-AP, Proteintech), and Ubiquitin (HY-P80925, MedChemExpress). Signals were detected using the ChemiDocMP imager (Bio-Rad).

RIPA lysis buffer containing protease inhibitor cocktails was used to lyse cells. After preclearing with Dynabeads protein G (10004D, Invitrogen), the cell lysis was immunoprecipitated with corresponding antibodies overnight at 4°C. The lysates were incubated at 4°C for an additional 2 h with the addition of beads. Prior to SDS-PAGE immunoblotting (IB), beads were washed three times with cold RIPA buffer and boiling at 100°C for 10 min.

### 2.4. Immunohistochemistry (IHC)

In the paper published before, the complete protocol was described [35]. In this study, the primary antibodies used for IHC included GPR34 (1:100, YT2014, Immunoway), USP8 (1:100, 67321-1-Ig, Proteintech), 4-HNE (1:75, ab46545, Abcam), and Ki-67 (2 μg/mL, 790-4286, Roche). Manling Zheng, an immunopathology technician at Department of Pathology, Zhuzhou Central Hospital verified the ATC biopsies.

### 2.5. Cell Proliferation, Colony Formation, and Migration Assays

According to manufacturer's instructions, the CCK8 kit (TP1197, Topscience) was used for evaluating cell viability. A colony formation experiment used 1000 cells per well seeded in a six-well plate, which were fixed with methanol and stained with 0.5% crystal violet after 2 weeks. For migration assays, 3 × 10^4^ cells were planted in a chamber. A layer of cells lining the chamber's upper surface was eliminated after 24 h, and the chamber was preserved with methanol before being stained with 0.5% cryostat.

### 2.6. Nude Mouse and Study Approval

All animal experiments were conducted in accordance with the protocols approved by the Ethnic Committee of Xiangya Hospital. The 4-week-old female nude mouse with optimal immunodeficiency and rapid tumor growth kinetics were purchased from Hunan SJA Laboratory Animal Co., Ltd. 5 × 10^6^ BHT101 cells were injected into the right underarm of each mouse, and every 3 days, tumor sizes were measured. Seventeen days after injection, euthanasia was performed on the mouse, and tumors were measured, weighed, and photographed.

### 2.7. Cell Imaging of Ferroptosis

In 12-well plates, 2 × 10^5^ BHT101 or 8305C cells were planted in each well and incubated with 5 or 2 µM RSL-3 for 18 h, respectively,and a microscope was used to capture the images.

### 2.8. Measurement of Lipid ROS and Total Glutathione (GSH) and Iron Assay

The complete protocols for the lipid ROS assay and total GSH detection were presented in the previous manuscript [36]. To measure lipid ROS, cells were treated with 10 µM C11-BODIPY (D3861, Thermo Fisher),and the GSH and GSSG Assay Kit (S0053, Beyotime) was used for detecting total GSH.

### 2.9. In Vivo Deubiquitination Assay

In vivo deubiquitination assay was conducted in BHT101 and 8305C cells with USP8 overexpression. To separate ubiquitinated GPR34, cells were treated with 10 µM MG132 (Selleck, S2619) for 12 h, followed by immunoprecipitation. Then, the anti-Ub antibody was used to detect the ubiquitination level of GPR34.

## 3. Statistical Analyses

With the exception of experiments involving nude mouse, all experiments have been repeated a minimum of three times. For statistical analysis, GraphPad Prism 8.0 and Microsoft Excel were used. Unless otherwise stated, Student's *t* test and analysis of variance (ANOVA) were used to compare two or more groups, respectively. Statistical significance was determined by a *p*-value less than 0.05. *⁣*^*∗*^ indicated *p*  < 0.05, *⁣*^*∗∗*^ indicated *p*  < 0.01, *⁣*^*∗∗∗*^ indicated *p*  < 0.001, and *⁣*^*∗∗∗∗*^ indicated *p*  < 0.0001.

## 4. Results

### 4.1. GPR34 Is Highly Expressed in ATC

**Figure 1:**
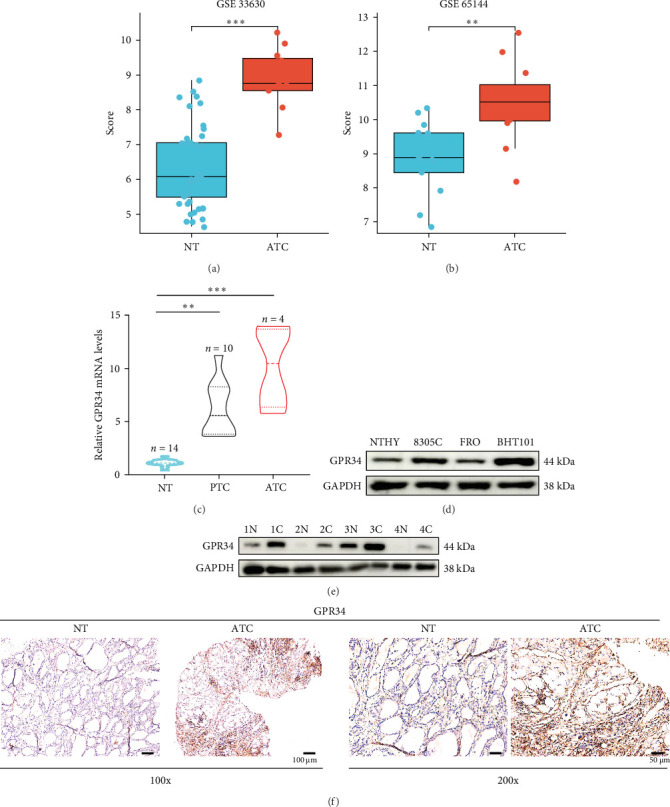
Aberrantly upregulated expression of GPR34 in ATC. (A, B) GPR34 mRNA expression level in NT and ATC tissues in the GSE 33,630 (A) and GSE 65,144 (B) databases. (C) GPR34 mRNA level was verified in the Zhuzhou Central Hospital THCA cohort including NT (*n* = 14), PTC (*n* = 10), and ATC (*n* = 4). (D) Western blot showed GPR34 protein level in normal thyroid and ATC cell lines. (E) Western blot showed GPR34 protein level in four pairs of NT and ATC tissues. (F) Representative IHC staining of GPR34 in NT and ATC tissues. *⁣*^*∗∗*^*p*  < 0.01, *⁣*^*∗∗∗*^*p*  < 0.001.

In order to study the role of GPR34 in ATC, we initially conducted GSE 33630 (NT [nontumor controls] = 45, ATC = 11) (Figure 1A) and GSE 65144 (NT = 13, ATC = 12) (Figure 1B) datasets to find that GPR34 mRNA level increased significantly in ATC. Additionally, examination of 14 clinical tissue pairs demonstrated that GPR34 exhibited higher mRNA levels in THCA, especially ATC, compared to NT (Figure 1C). To further investigate the protein levels of GPR34 in ATC, we first applied HPA dataset to reveal that GPR34 was higher in THCA compared to NT (Figure S1A). Then, we assessed GPR34 expression in ATC cell lines and the normal thyroid cell line NTHY-ORI 3-1, the results indicated that GPR34 was elevated in ATC cell lines (Figure 1D). In addition, we found that GPR34 was also considerably greater in clinical ATC tissues than NT (Figure 1E). Moreover, IHC analysis presented that ATC cells exhibited strong staining for GPR34 in comparison to NT cells (Figure 1F). The survival analysis showed that high GPR34 expression was positively correlated with improved survival in thyroid cancer patients (Figure S1B). Taken together, these data implied that GPR34 could be a potential diagnostic marker for ATC.

### 4.2. GPR34 Deletion Suppresses ATC Progression

**Figure 2:**
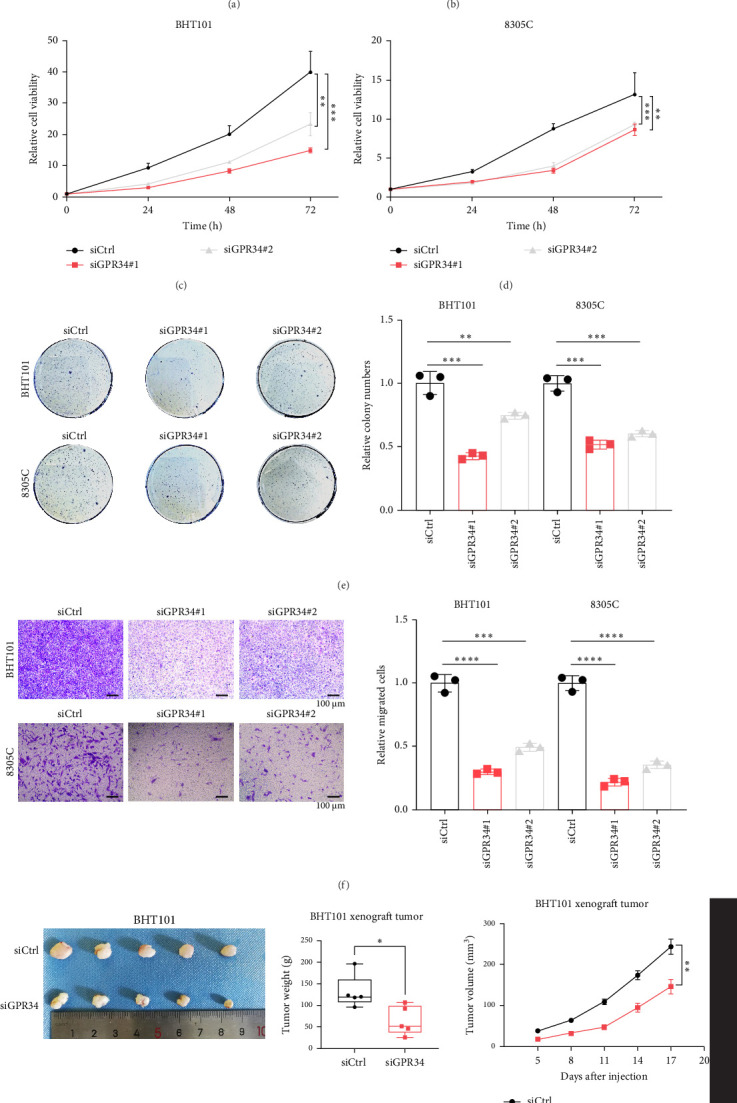
GPR34 silencing suppresses ATC progression. (A, B) Western blot showed siGPR34#1 and siGPR34#2 transfection efficiency in BHT101 (A) and 8305C cells (B). (C, D) The CCK8 assay represented GPR34 knockdown suppressed the proliferation of BHT101 (C) and 8305C (D) cells. (E) The colony formation assay of ATC cells with GPR34 deletion. (F) The transwell migration experiment indicated that GPR34 silencing decreased the migration capability of ATC cells. (G) BHT101 cells transfected with indicated plasmids were transplanted on nude mouse (*n* = 5 mouse per group). The tumors were removed and weighted after 17 days. The tumor size was assessed every 3 days. *⁣*^*∗*^*p*  < 0.05, *⁣*^*∗∗*^*p*  < 0.01, *⁣*^*∗∗∗*^*p*  < 0.001, *⁣*^*∗∗∗∗*^*p*  < 0.0001.

To further evaluate the effect of GPR34 on ATC progression, we knocked down GPR34 in BHT101 and 8305C cells with high GPR34 expression (Figure 2A,B, Figure S2A). The deletion of GPR34 obviously decreased the proliferation and colony formation of ATC cells revealed by CCK8 analysis (Figure 2C,D) and colony formation experiments (Figure 2E). The transwell migration assay showed that GPR34 silencing significantly suppressed the migration ability of ATC cells (Figure 2F). Moreover, we examined whether GPR34 knockdown could suppress ATC growth in a xenograft mouse model. The in vivo experiment further corroborated that GPR34 suppression markedly impeded tumor growth (Figure 2G, Figure S2B,C). Together, our findings established GPR34 as a crucial regulator in sustaining the malignant phenotypes of ATC.

### 4.3. GPR34 Silencing Confers Ferroptosis of ATC

**Figure 3:**
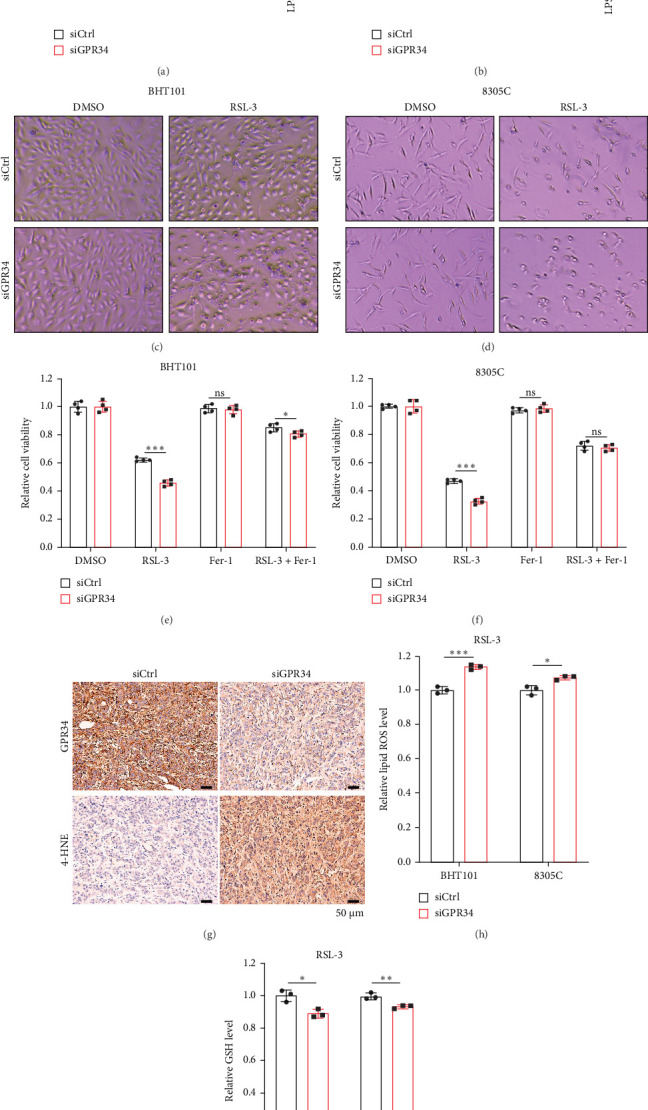
GPR34 deletion promotes ferroptosis of ATC cells. (A, B) The CCK8 assay was used to determine the viability of BHT101 (A) and 8305C (B) cells with GPR34 knockdown after addition of the apoptosis inducer VCR, ferroptosis inducer RSL-3, autophagy inducer rapamycin, or pyroptosis inducer LPS + nigericin. (C, D) Representative images were presented of BHT101 (C) and 8305C cells (D) with GPR34 deletion treated with RSL-3. (E, F) The CCK8 assay was used to assess the responses of BHT101 (E) and 8305C cells (F) with GPR34 silencing to RSL-3 ± Fer-1. (G) IHC test on xenograft tumors was performed to identify the correlation between GPR34 and 4-HNE expression. (H, I) Lipid ROS (H) and GSH (I) levels in GPR34 deletion ATC cells. Ns, nonsignificant. *p*  > 0.05, *⁣*^*∗*^*p*  < 0.05, *⁣*^*∗∗*^*p*  < 0.01, *⁣*^*∗∗∗*^*p*  < 0.001.

Given the substantial influence of GPR34 on apoptosis [26, 30, 31], it is of considerable interest to explore the effect of GPR34 deletion on the cell fate of ATC cells. Consequently, we evaluated the therapeutic efficacy of several pharmaceuticals including the apoptosis inducer VCR, the ferroptosis inducer RSL-3, the autophagy inducer rapamycin, or the pyroptosis inducer LPS + nigericin, respectively. We found that ferroptosis inducer RSL-3 exhibited a pronounced efficacy in inducing cell death relative to other inducers (Figure 3A,B). Then, after treating GPR34 deletion BHT101 and 8305C cells with RSL-3, the morphological evidence indicated that GPR34 knockdown enhanced ferroptosis (Figure 3C,D). Furthermore, we analyzed the RSL-3-induced ferroptosis in the presence or absence of ferrostatin, a ferroptosis inhibitor, to prove that GPR34 silencing had a remarkable impact on the growth inhibition of ATC cells induced by RSL-3 (Figure 3E,F). Moreover, IHC assays on xenograft tumors showed that GPR34 knockdown obviously increased 4-HNE expression, a recognized biomarker of ferroptosis (Figure 3G). Additionally, we determined that GPR34 suppression led to an increase in intracellular lipid ROS (Figure 3H) and a concomitant decrease in total GSH (Figure 3I). At last, GEPIA analysis of TCGA-THCA revealed positive correlations between GPR34 and ferroptosis suppressors NFE2L2 and SCD (Figure S3A, B), and SCD mRNA levels significantly decreased in GPR34 knockdown ATC cells (Figure S3C,D). All together, these findings indicated that GPR34 deletion facilitated the ferroptosis of ATC.

### 4.4. USP8 Stabilizes GPR34 Through Deubiquitination

**Figure 4:**
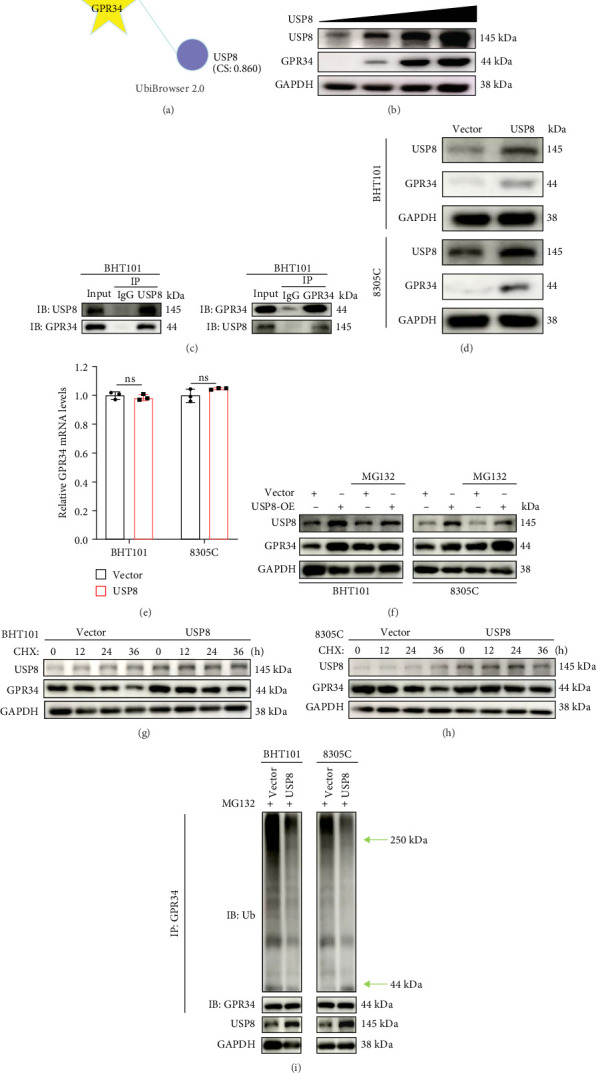
USP8 interacts with and deubiquitinates GPR34. (A) Predicted deubiquitinating enzymes of GPR34 by UbiBrowser 2.0. (B) Following transfection of BHT101 cells with increasing amounts of USP8 WT plasmids, western blot was carried out to detect GPR34 expression. (C) Co-IP and immunoblotting (IB) were used to analyze the endogenous interaction between USP8 and GPR34. Immunoprecipitated BHT101 cells were followed by IB with anti-USP8 or anti-GPR34 antibody. (D) The GPR34 level in USP8 overexpressed ATC cells was detected by Western blot. (E) Analysis of the GPR34 mRNA level in ATC cells with USP8 overexpression. (F) ATC cells overexpressing USP8 were treated with or without MG132, and GPR34 protein levels were measured by Western blot. (G, H) CHX was applied to BHT101 (G) and 8305C (H) cells overexpressing USP8 for the indicated duration, followed by Western blot. (I) BHT101 cells overexpressing USP8 were treated with MG132 before collection. Anti-GPR34 and anti-Ub antibodies were employed for the immunoprecipitation and immunoblotting of GPR34. Ns, nonsignificant. *p*  > 0.05.

To explore the potential deubiquitinating enzymes associated with GPR34, we first utilized UbiBrowser 2.0, which identified USP8 (confidence score [CS] = 0.860) and USP33 (CS = 0.813) as high-confidence interactors with GPR34 (Figure 4A). Subsequently, we transfected BHT101 cells with increased amounts of USP8 or USP33 to identify that USP8 overexpression elevated GPR34 expression significantly (Figure 4B), but not USP33 (Figure S4A). In addition, we further used Co-IP experiment to demonstrate that USP8 endogenously coimmunoprecipitated GPR34 in BHT101 cells (Figure 4C). USP8 expression was also elevated in ATC showed by IHC assays (Figure S4B). To further analyze whether USP8 regulated GPR34 via ub-proteasome pathway, we overexpressed USP8 in BHT101 and 8305C cells to indicate that overexpression of USP8 increased GPR34 protein level (Figure 4D), but did not affect GPR34 mRNA expression (Figure 4E), and this increase could be rescued by proteasome inhibitor MG132 (Figure 4F). Additionally, MG132 treatment partially reverses GPR34 reduction induced by USP8 knockdown in both BHT101 and 8305C cell lines (Figure S4C). Moreover, cycloheximide (CHX) chase assays were conducted to show that the half-life of GPR34 was extended in ATC cell with USP8 overexpression (Figure 4G,H, Figure S4D,E). Due to USP8′s role as a deubiquitinating enzyme, we further examined whether USP8 directly deubiquitylated GPR34. As depicted in Figure 4I, the ubiquitin on GPR34 protein dramatically decreased in USP8 overexpressed cells. In summary, these results demonstrated that USP8 could stabilize GPR34 through deubiquitination.

### 4.5. USP8 Reverses the Effects of GPR34 Suppression

**Figure 5:**
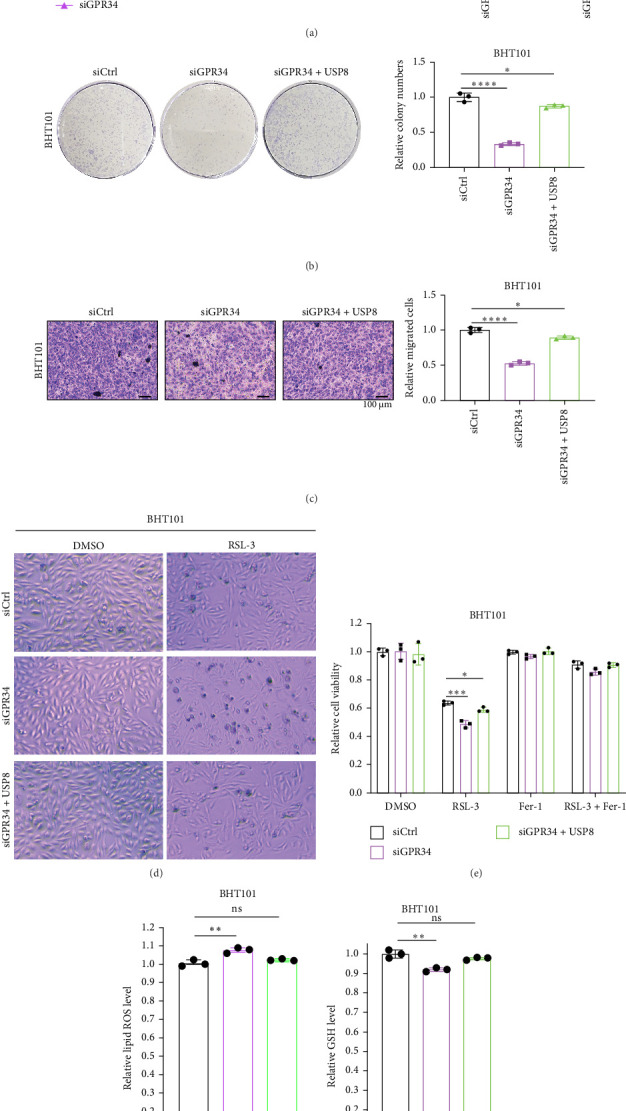
Increased USP8 expression reverses the effect of GPR34 knockdown. (A) CCK8 analysis presented that USP8 reversed GPR34 deletion-induced inhibition of proliferation in BHT101 cells. (B) USP8 overexpression increased the ability of BHT101 cells with GPR34 knockdown to form clones based on colony formation assay results. (C) Transwell migration experiment of BHT101 cells deletion of GPR34 with USP8 overexpression. (D) Representative images of GPR34 deletion BHT101 cells with USP8 overexpression treated with RSL-3. (E) The CCK8 assay determined the responses of BHT101 cells deletion of GPR34 with USP8 overexpression to RSL-3 ± Fer-1 (DMSO-treated groups are normalized to 1.0 for comparative analysis of drug responses). (F, G) In GPR34 deletion BHT101 cells with USP8 overexpression, lipid ROS (F), and GSH (G) levels were measured. Ns, nonsignificant. *p*  > 0.05, *⁣*^*∗*^*p*  < 0.05, *⁣*^*∗∗*^*p*  < 0.01, *⁣*^*∗∗∗*^*p*  < 0.001, *⁣*^*∗∗∗∗*^*p*  < 0.0001.

To investigate whether the carcinogenic ability of GPR34 was modulated by USP8, we further conducted rescue experiments in GPR34 knockdown BHT101 cells with USP8 overexpression. The results from the biological function experiments indicated that USP8 elevation remarkably ameliorated the reduced cell viability (Figure 5A), diminished colony formation (Figure 5B), and impaired migratory capacity (Figure 5C) induced by GPR34 suppression. Furthermore, we identified that overexpressing USP8 counteracted the ferroptosis of BHT101 cells caused by GPR34 deletion (Figure 5D, E). Consistently, increased intracellular lipid ROS (Figure 5F) and decreased GSH (Figure 5G) generated by GPR34 silencing could be mitigated by elevating USP8. Additionally, USP8-driven proliferation (Figure S5A) and ferroptosis resistance (Figure S5B) were partially rescued by GPR34 silencing. Generally, GPR34 is the essential downstream mediator of USP8-driven oncogenesis and ferroptosis resistance in ATC.

### 4.6. DUB-IN-3 Inhibits ATC Progression by Targeting USP8 to Promote Ferroptosis

**Figure 6:**
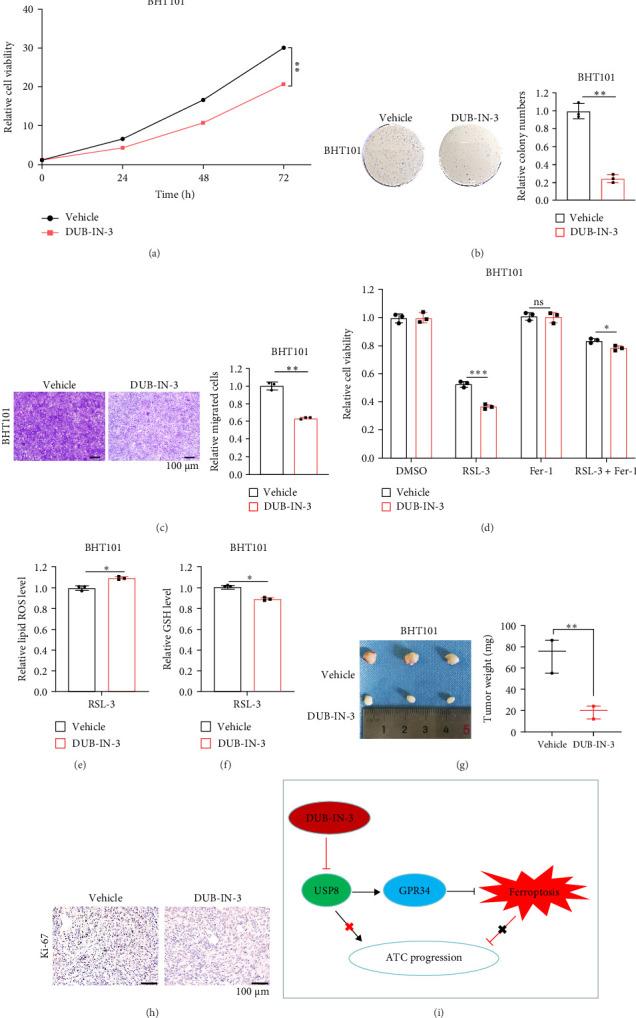
DUB-IN-3 inhibits ATC progression by activating ferroptosis. (A-C) The CCK8 assay (A), colony formation assay (B), and transwell assay (C) was applied to present the effect of DUB-IN-3 on the proliferation, colony formation, and migration capability of BHT101 cells. (D) The CCK8 assay was used to assess the responses of BHT101 cells with DUB-IN-3 treatment to RSL-3 ± Fer-1. (E, F) Lipid ROS (E) and GSH (F) levels in DUB-IN-3 treating ATC cells. (G) The parental BHT101 cells were injected into the right underarm of nude mouse, then DUB-IN-3 was intraperitoneally injected every 3 days (*n* = 3 mouse per group). The tumors were removed and weighted after 17 days. (H) Representative images of IHC staining for Ki-67. (I) GPR34 stabilized by deubiquitinase USP8 promotes ATC progression via inhibiting ferroptosis. Ns, nonsignificant. *p*  > 0.05, *⁣*^*∗*^*p*  < 0.05, *⁣*^*∗∗*^*p*  < 0.01, *⁣*^*∗∗∗*^*p*  < 0.001.

We used the USP8 inhibitor DUB-IN-3 to further explore the possibility of targeting USP8 to improve the efficacy of ATC therapy. Notably, suppression of USP8 by DUB-IN-3 significantly reduced the proliferation ability of BHT101 cells as judged by cell viability assay (Figure 6A) and colony formation experiment (Figure 6B). Additionally, treatment of DUB-IN-3 resulted in a remarkable migration arrest of BHT101 cells (Figure 6C). Furthermore, DUB-IN-3 dose-dependently induced ferroptosis, as evidenced by synergized cell death with RSL-3 (Figure 6D), elevated lipid ROS (Figure 6E), and depleted GSH (Figure 6F). Moreover, the xenograft nude mouse model further proved that inhibition of USP8 by DUB-IN-3 could suppress ATC growth in vivo (Figure 6G, H). Collectively, the above data revealed that targeting USP8 represses ATC progression by activating ferroptosis.

## 5. Discussion

ATC is a rare THCA subtype featured with local aggressiveness, high rate of metastasis, and rapid fatal prognosis [37]. Thus, it is urgent to identify novel candidate molecules that can be targeted for ATC therapy. GPR34, considered as orphan receptors, has been demonstrated as a crucial cancer driver in various cancers, including cervical cancer, gastric cancer, and glioma [24, 28, 38]. However, whether GPR34 is a potential target for improving ATC treatment remains undetermined. In the present study, we conducted a comprehensive investigation into the expression level, impacts on tumorigenesis and PCD, and corresponding DUB of GPR34 in ATC.

GPR34, a member of the G protein-coupled receptor (GPCR) superfamily, plays a crucial role in fundamental biological processes, including cellular growth, cell metabolism, and gene transcription [22, 39]. Multiple evidence have indicated that GPR34 has elevated expression level in malignancies, such as gastric cancer, glioma, TNBC, and MALT lymphoma, and contributes to the pathogenesis and progression of these cancers [23, 26–28], while the expression level and oncogenic role of GPR34 in ATC remain poorly understood. Here, we first employed GEO datasets to show that GPR34 significantly increased in ATC tissues. Subsequently, this was further confirmed by RT-qPCR, western blot, and IHC staining of clinical ATC tissues. Furthermore, we demonstrated that GPR34 knockdown obviously repressed the proliferation, colony formation, and migration of ATC cell, a result that was further validated in vivo using xenografted nude mouse models. Notably, while our study demonstrates that GPR34 drives ATC progression, public cohort data indicate its association with favorable prognosis in general THCA. This divergence may reflect the high heterogeneity of THCA subtypes, wherein GPR34′s function could vary across contexts. Collectively, GPR34 is identified as being highly expressed and implicated in ATC progression for the first time.

PCD, encompassing apoptosis, autophagy, pyroptosis, ferroptosis, and necroptosis, are evolutionarily conserved cellular suicide mechanisms regulated by multiple signaling pathways that plays an important role in many biological processes and contribute to carcinogenesis and cancer progression [14, 40]. Emerging studies determines that GPR34 is involved in inhibiting apoptosis, and the Q340X truncation of GPR34 confers a significantly increased resistance to apoptosis [26, 30], but the specific role of GPR34 in other forms of PCD remains largely unexplored. In this study, we first investigated the effect of GPR34 knockdown on PCD by employing various pharmacological agents to induce PCD, and the results showed that the ATC cells with or without GPR34 deletion had the greatest difference in cell viability when exposed to the ferroptosis inducer RSL-3. Then, we analyzed the RSL-3-induced ferroptosis in the presence or absence of ferrostatin to further substantiate that GPR34 exerted a significant inhibitory effect on ferroptosis. IHC staining indicated a negative correlation between the ferroptosis marker 4-HNE and GPR34 expression. Moreover, GPR34 silencing increased the lipid ROS level while decreased total GSH. Overall, this is the first study to clarify that GPR34 is a critical suppressor of ferroptosis and may be a potential target for the elimination of ATC cells.

GPCRs comprise the largest and most diverse family of signal-mediated receptors which affect a vast array of biological and physiological cell functions, and contribute to the progression and metastasis of multiple human cancers [41, 42]. Modulating GPCRs signaling is critical for the treatment of various cancers, and the posttranslational modifications including deubiquitination have been demonstrated to be integral to the regulation of GPCR function [32]. Although growing studies shows that DUBs including USP4, USP14 and USP24 play a significant role in stabilizing GPCRs such as A2AR, CXCR4, and GPR31, the specific DUBs responsible for GPR34 remains unknown [33, 43, 44]. In the present study, we first applied bioinformatics analysis to identify a high coincidence score for the interaction between USP8 or USP33 with GPR34. Then, experiments of increasing plasmid amount and Co-IP assay indicated that GPR34 only had physiological interaction with USP8. Additionally, overexpression of USP8 significantly increased GPR34 protein level without affecting its mRNA expression, and this promotion could be rescued by MG132. Furthermore, the CHX chase assays determined that the half-life of GPR34 was obviously prolonged by USP8 overexpression. Moreover, the deubiquitinating assays demonstrated that USP8 was capable of removing ubiquitin chains from the GPR34 protein. Afterward, we further conducted rescue experiments to show that the growth suppression and ferroptosis promotion induced by GPR34 deletion could be reversed by USP8 overexpression. Generally, USP8 could deubiquitylates GPR34, thereby facilitating ATC progression by inhibiting ferroptosis.

USP8, belonging to the USP protease family, is increased in multiple kinds of cancers, including melanoma, gastric cancer, liver cancer, and lung cancer. The upregulation of USP8 expression is generally correlated with poor prognosis and malignant progression of tumors [45–48]. Additionally, several studies indicate that USP8 confers ferroptosis resistance through β-catenin stabilization or OGT-SLC7A11 axis in HCC [47], and inhibits ferroptosis via regulating OTUB1-SLC7A11 pathway in chronic obstructive pulmonary disease (COPD) [49]. Furthermore, it has been reported that targeting USP8 with DUB-IN-3, a specific inhibitor of USP8, could suppress the progression of HCC and intrahepatic cholangiocarcinoma (iCCA) [47, 50]. Considering the promoting effect of USP8 on ATC progression, we further explored whether DUB-IN-3 could mitigate the malignancy of ATC. This study demonstrated that DUB-IN-3 significantly inhibited the proliferation and migration of ATC cells, as evidenced by biological function experiments. Consistent with in vitro assays, the xenograft nude mouse model also indicated that DUB-IN-3 suppressed ATC progression. Additionally, DUB-IN-3 provoked ferroptosis in ATC, confirmed by the increased lipid ROS and reduced total GSH. In short, targeting USP8 with DUB-IN-3 significantly limits the malignant proliferation of ATC by activating ferroptosis.

In summary, we found that elevated GPR34 expression is associated with malignant behavior in ATC. Additionally, we revealed that GPR34 confers ferroptosis tolerance to promote ATC progression for the first time. Furthermore, we identified USP8 is a novel DUB of GPR34, which could rescue the effects caused by GPR34 deletion, and targeting USP8 with DUB-IN-3 could restrain tumor growth in ATC (Figure 6I), offering a new potential strategy for this treatment-resistant cancer. However, the precise molecular mechanism by which GPR34 modulates ferroptosis has not been detailed discussed in this study. It remains to be investigated whether the suppressive effect of GPR34 on ferroptosis is present in other cancer types.

## Data Availability

The materials or data used and analyzed during this study will be available from the corresponding author upon reasonable request.
