# Supplementary material for: GPR34 Stabilized by Deubiquitinase USP8 Suppresses Ferroptosis of ATC
Source: Mediators Inflamm. 2025 Aug 18;2025:5576056. doi: 10.1155/mi/5576056 (PMC12377955; doi:10.1155/mi/5576056)
Supplement: Supporting Information — More supporting information can be found online in the Supporting Information section. This file includes Figures S1 to S5 and Table S1. [file 5576056.f1.zip › Table S1.docx]

**Table S1. The sequences of the primers for RT-qPCR used in the present study.**

| Name | Forward premier (5’-3’) | Reverse premier (5’-3’) |
| --- | --- | --- |
| GPR34 | CTGGTTGGGAACATAATCGCCC | GGCAGAAGATGAGTAGGAGGTC |
| USP8 | GATCGTACCAGGACTGCCTTCA | GCAGATGAAGGAGCCATTTCCG |
| GAPDH | GTCTCCTCTGACTTCAACAGCG | ACCACCCTGTTGCTGTAGCCAA |
| NFE2L2 | CACATCCAGTCAGAAACCAGTGG | GGAATGTCTGCGCCAAAAGCTG |
| SCD | CCTGGTTTCACTTGGAGCTGTG | TGTGGTGAAGTTGATGTGCCAGC |
