# Supplementary figures and images for: GPR34 Stabilized by Deubiquitinase USP8 Suppresses Ferroptosis of ATC
Source: Mediators Inflamm. 2025 Aug 18;2025:5576056. doi: 10.1155/mi/5576056 (PMC12377955; doi:10.1155/mi/5576056)

A

GPR34

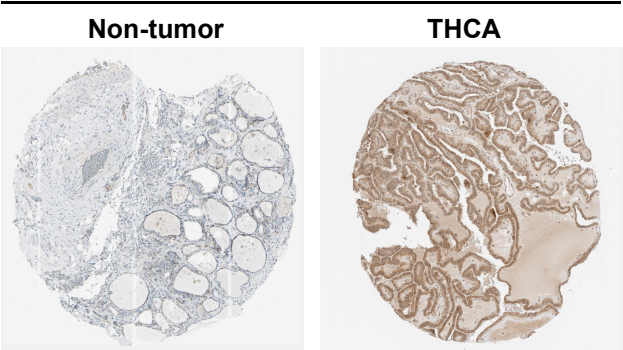

B

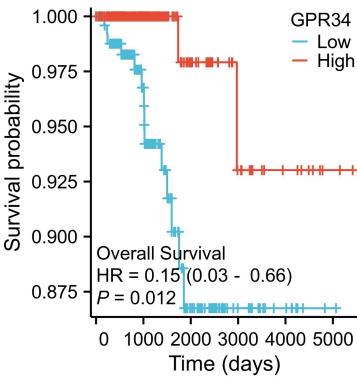

Supplement: Supporting Information — More supporting information can be found online in the Supporting Information section. This file includes Figures S1 to S5 and Table S1. [file 5576056.f1.zip › Fig.S1.pdf]

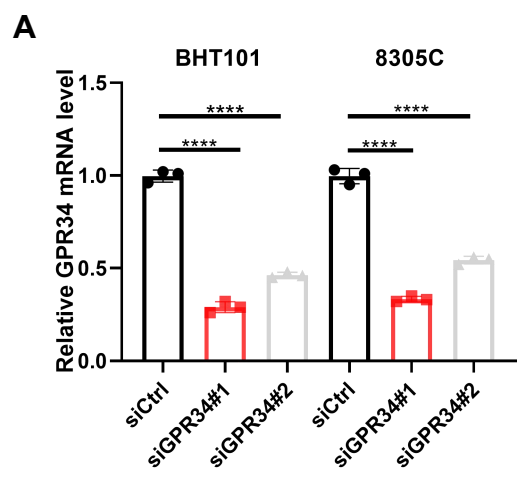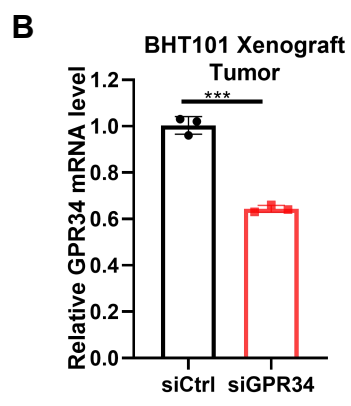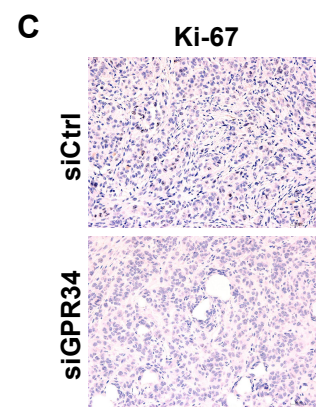

Supplement: Supporting Information — More supporting information can be found online in the Supporting Information section. This file includes Figures S1 to S5 and Table S1. [file 5576056.f1.zip › Fig.S2.pdf]

**A**

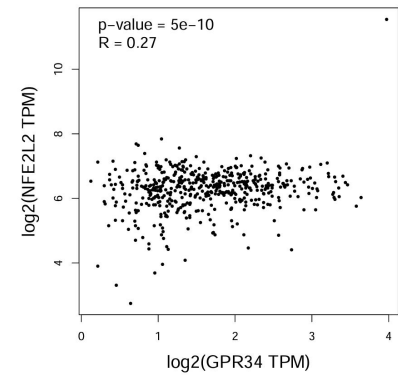

**B**

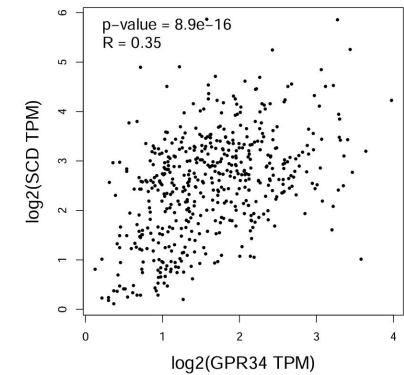

**C**

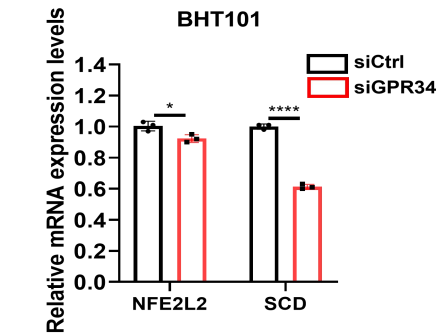

**D**

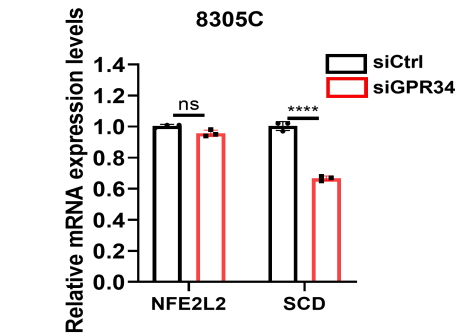

Supplement: Supporting Information — More supporting information can be found online in the Supporting Information section. This file includes Figures S1 to S5 and Table S1. [file 5576056.f1.zip › Fig.S3.pdf]

**A**

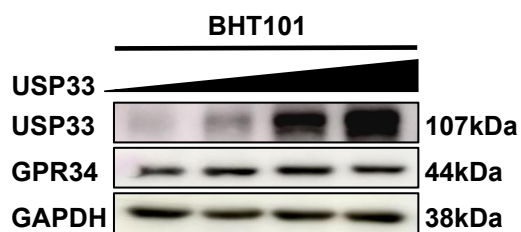

**B**

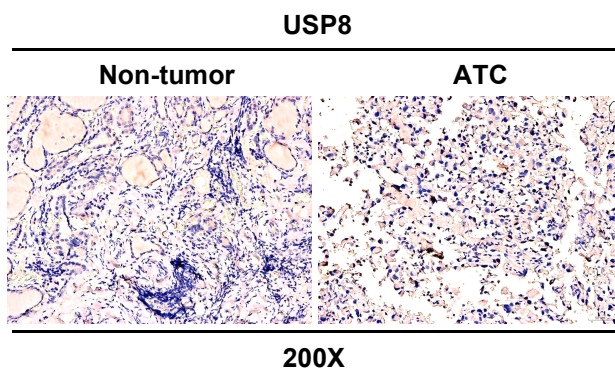

**C**

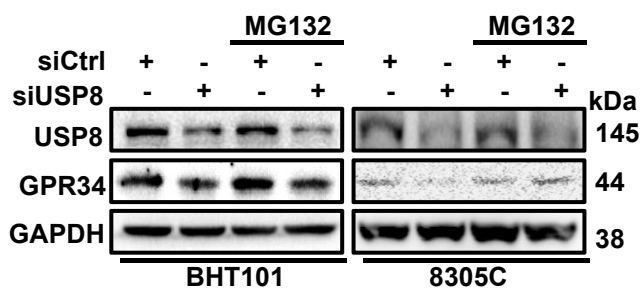

**D**

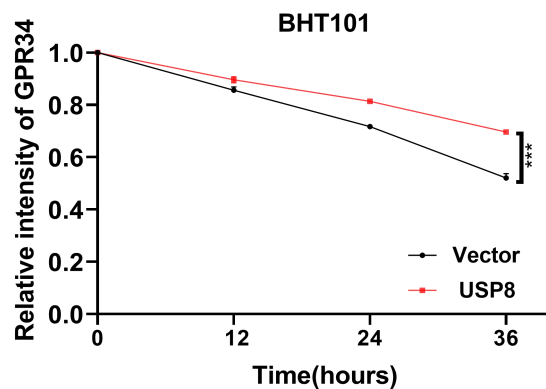

**E**

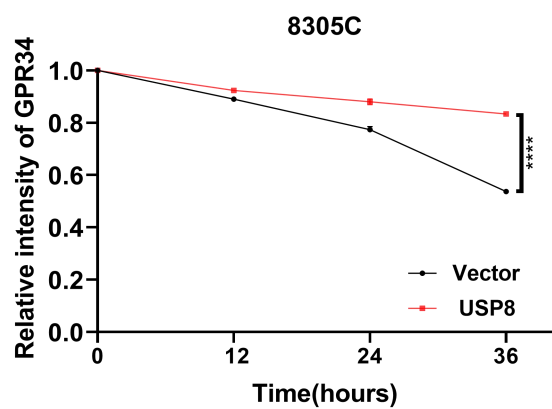

Supplement: Supporting Information — More supporting information can be found online in the Supporting Information section. This file includes Figures S1 to S5 and Table S1. [file 5576056.f1.zip › Fig.S4.pdf]

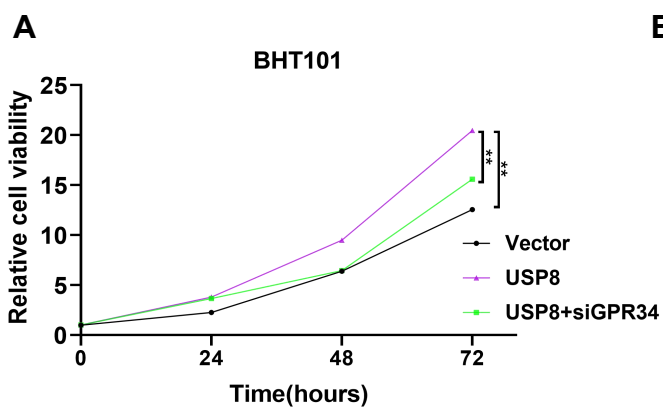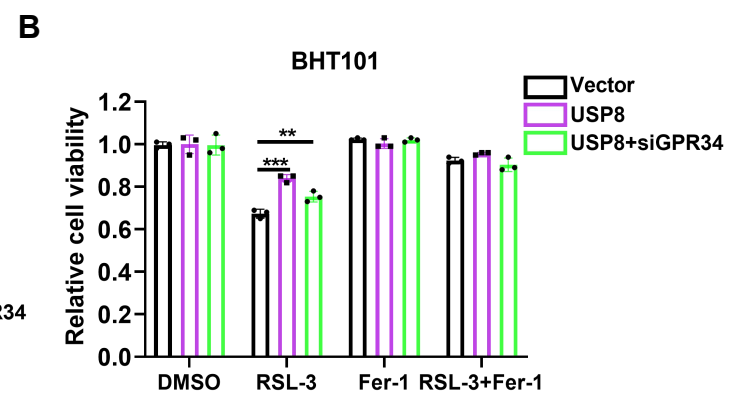

Supplement: Supporting Information — More supporting information can be found online in the Supporting Information section. This file includes Figures S1 to S5 and Table S1. [file 5576056.f1.zip › Fig.S5.pdf]
